# The Impact of Fracture Incidence on Health Related Quality of Life among Community-Based Postmenopausal Women

**DOI:** 10.1155/2015/717914

**Published:** 2015-07-30

**Authors:** A. L. Barcenilla-Wong, J. S. Chen, M. J. Cross, L. M. March

**Affiliations:** Institute of Bone and Joint Research, Kolling Institute of Medical Research, University of Sydney, Sydney, NSW 2065, Australia

## Abstract

This prospective study aimed to examine the impact of fracture incidence on health-related quality of life (HRQOL) among postmenopausal women. Study subjects were Australian female community-dwellers in the Global Longitudinal Study of Osteoporosis in Women (GLOW). Self-administered questionnaires were collected annually from 2007 to 2010. Outcomes were the Medical Outcomes Study Short Form-36 (SF-36 physical function (SF36PFS) and vitality (SF36VS) scores), European Quality of Life (EQ-5D), and self-reported general health (GH) of excellent/good. Questionnaires were divided into prior to, the 1st, the 2nd, and the 3rd year after incident fracture assessments. Generalized linear models with generalised estimating equations (GEE) were employed for the analysis. The 2,872 participants (age: median 65; interquartile range 60–73 years) provided a total of 10,436 assessments including 266, 165 and 76 assessments for the 1st, the 2nd, and the 3rd year after incident fracture, respectively. Multivariate adjustments showed reductions in HRQOL measures peaking at the 1st year for SF36VS (coefficient −3.0; 95% CI: −5.1, −0.8) and EQ-5D (coefficient −0.03; 95% CI: −0.06, −0.00) and at the 2nd year for SF36PFS (coefficient −3.0; 95% CI: −5.6, −0.5) and GH (odds ratio 0.92; 95% CI: 0.70, 1.19). Fracture incidence reduced HRQOL including vitality and physical function among relatively young, healthy postmenopausal women and the reduction in European Quality of Life measure was clinically important.

## 1. Introduction

Fractures associated with osteoporosis inflict considerable burden and disability worldwide [1–3]. The disease has also been shown to contribute to reductions in health-related quality of life (HRQOL), particularly following osteoporotic fractures of the hip and spine [4–6]. Despite past studies describing changes to HRQOL following a fracture, most have been limited to either cross-sectional analyses [7–9] or prospective studies predominantly focused on the effects of prevalent fractures [10]. Other prospective studies exploring incident fractures relied on retrospective recollections to provide baseline measures of HRQOL [11, 12] or measured baseline readings after a fracture had occurred [13].

Much can be gained in examining the true impact of incident fractures on HRQOL using data collected immediately prior to as well as in the years following an incident fracture. This approach, however, has not been utilised extensively in the past. The purpose of this study is to use longitudinal data to examine the impact of fracture incidence on HRQOL among community-based postmenopausal women.

## 2. Methods

### 2.1. GLOW Participants and Recruitment

The Global Longitudinal Study of Osteoporosis in Women (GLOW) is a prospective, observational cohort study recruiting over 60,000 women ≥55 years from 10 countries, including Australia. Details of the study have been described previously [14, 15].

### 2.2. Study Subjects

Between January 2007 and February 2008, a convenience sample of 8,029 eligible female Australian patients was identified through 51 general practitioners from 14 Sydney-based practices. General practitioners invited eligible patients to participate in the study through a mailed GLOW information packet containing study information, a participant consent form, and a reply-paid envelope. Consenting participants (*n* = 3,011) were then mailed the self-administered baseline GLOW questionnaire and a reply-paid envelope for return. The Australian baseline GLOW study sample consisted of 2,904 participants (age range 55–96 years) who completed and returned the baseline questionnaire. Annual questionnaires were sent between 2007 and 2010 and attained a final follow-up response rate of 95%. Analyses were conducted among 2,872 Australian postmenopausal community-dwellers in GLOW after excluding women who reported more than one incident fracture (*n* = 32) during the study period. The study was approved by the Northern Sydney Human Research Ethics Committee.

### 2.3. Questionnaires

Self-administered questionnaires were mailed annually from 2007 to 2010. Full details regarding the questionnaire have been previously described [14]. In brief, the questionnaire contained previously published validated instruments including the Medical Outcomes Study Short Form-36 (SF-36) and European Quality of Life (EQ-5D). The questionnaires explored various aspects of bone health including patient characteristics, risk factors, use of medications, perception of risks, and health care use.

The main outcome measures included SF-36 physical function (SF36PFS) and vitality (SF36VS) scores, EQ-5D (descriptive portion), and general health (GH) of excellent/good. A higher score in each SF-36 subscale equated to better HRQOL. A five-point difference on the (0–100 scale) has been considered to be the minimal important difference in SF-36 subscales [16]. An EQ-5D utility score of 1.00 represents full health and 0.00 is equivalent to death. For those with osteoporosis, the minimal clinically important difference in EQ-5D scores is 0.03 [17]. Questionnaires were divided into prior to, the 1st, the 2nd, and the 3rd year after incident fracture assessments. Incident fracture occurrence and location were based on self-report.

Other information collected included age at assessment, education level, private health insurance status, body mass index (BMI), smoking status, alcohol drinking, comorbidities (including asthma, chronic bronchitis/emphysema, osteoarthritis/degenerative joint disease, rheumatoid arthritis, high cholesterol, hypertension, or osteoporosis), previous fracture since the age of 45 years, number of falls in the previous year, prior year weight loss (>5 kilograms), and self-reported use of antiosteoporosis medications (AOM) (i.e., oestrogen, selective oestrogen receptor modulators, bisphosphonates, calcitonin, parathyroid hormone, strontium, calcium, and vitamin D). Information on treatment sought after an incident fracture (including visits to a doctor's office or clinic, hospital visit/stays, surgery following fracture, rehabilitation, and/or nursing homes stays) was also collected.

### 2.4. Statistical Analysis

Descriptive statistics were used to summarize baseline (first assessment) characteristics of the study women by incident fracture during the study period. Characteristic differences between women with and without incident fracture were assessed using the chi-square test or analysis of variance where appropriate. Generalized linear regression models were employed to determine whether there were statistically significant differences (i.e., two-sided* P* value ≤ 0.05) in study outcomes between prior to incident fracture assessments and the 1st, the 2nd, and the 3rd year postincident fracture assessments. The coefficients or odds ratios (OR) plus 95% confident intervals (CI) were reported for the 1st, the 2nd, and the 3rd year after incident fracture assessments using prior incident fracture assessment as the reference group. Lack of independence in study outcomes for multiple assessments in the same woman was taken into account using generalised estimating equations (GEE). Multivariable regression analyses were also conducted allowing for adjustment of potential confounders including age, private insurance, tertiary education, history of prior fracture after 45 years, body mass index, prior year weight loss ≥5 kgs, any comorbidity, current smoking, drinking ≥7 drinks/week, and number of falls in the prior year. These factors were included a priori from past literature that had been shown to influence the HRQOL outcomes as well as those factors that were significant at the 95% level in the univariate analyses. Subanalyses excluding single region fractures of the hand, foot, digital, and toe were also performed. Student's paired *t*-tests were performed to determine changes to HRQOL measures between baseline and both 1-year and 2-year follow-up results, according to incident fracture site. All analyses were performed using SAS 9.3.

## 3. Results

The 2,872 participants (age: median 65; interquartile range 60–73 years) provided a total of 10,346 assessments including 266 assessments in the 1st year, 165 assessments in the 2nd year, and 76 assessments in the 3rd year following an incident fracture.  Table 1 shows baseline (first assessment) characteristics of the study participants. A greater number of participants who had an incident fracture during follow-up were smokers (7.9% versus 4.5%), had comorbidities (69.9% versus 60.7%), and were more likely to have at least one fall in the prior year compared to participants without an incident fracture. Those with incident fractures were also significantly more likely to have had a prior fracture after the age of 45 (37.5% versus 23.5%) and were taking AOM (18.1% versus 10.0%) prior to an incident fracture occurring. There was no significant difference between age, BMI, private health insurance status, tertiary education, and prior year weight loss between the two groups.

In total, there were 266 self-reported fracture incidences for this study including 238 single region fractures and 28 multiple regions fractures. Single region incident fractures occurring in the lower leg region (foot, ankle, lower leg, knee, and upper leg) were the most reported (*n* = 65), followed by the upper limb region (hand, wrist, arm, and elbow) (*n* = 55), the finger/toe (*n* = 52), rib (*n* = 28), spine (*n* = 20), shoulder/clavicle (*n* = 7), hip (*n* = 6), and pelvis (*n* = 5).

Due to the small number of fractures in each location (particularly in commonly studied osteoporotic fracture sites such as the hip and spine) and many fracture cases of multiple regions affected in a single incident, the following analyses were conducted using any fracture incidence or event as the study unit.

### 3.1. Analyses Including All Fracture Locations

Univariate analyses that included all fracture locations revealed significant reductions in HRQOL measures (Table 2). SF36PFS reduced significantly until 2 years after fracture where the reduction also became clinically significant (coefficient −5.0; 95% CI: −7.6, −2.3). Significant reductions in SF36VS (coefficient −4.1; 95% CI: −6.0, −2.1) and a clinically significant reduction in EQ-5D (coefficient −0.04; 95% CI: −0.07, −0.02) were seen only at 1 year after incident fracture. At the 2nd and the 3rd year after fracture, both SF36VS and EQ-5D remained lower than the baseline values but the reductions were not statistically significant. Those with an incident fracture were less likely to report having an “excellent/very good/good” GH rating compared to those without an incident fracture. This was, however, only significant at 1 year after fracture (odds ratio 0.80; 95% CI: 0.67, 0.96).

Multivariate adjustments for age at assessment, insurance status, education level, history of prior fracture after the age of 45 years, body mass index, prior year weight loss (≥5 kilograms), any comorbidity, current smoking, alcohol drinking (≥7 drinks/week), and number of falls in the prior year revealed significant reductions to SF36PFS (coefficient −3.0; 95% CI: −5.6, −0.5) at 2 years after incident fracture (Table 2). Statistically significant reductions in SF36VS (coefficient −3.0; 95% CI: −5.1, −0.8) and a clinically significant reduction in EQ-5D (coefficient −0.03; 95% CI: −0.06, −0.00) were seen only at 1 year following an incident fracture (Table 2). There was no significant difference in “excellent/very good/good” GH rating in those with or without an incident fracture (coefficient 0.97; 95% CI: 0.78, 1.21).

### 3.2. Analyses Excluding Single Region Hand, Foot, Digital, and Toe Fractures

Univariate and multivariate analyses conducted excluding (*n* = 66) single region fractures of the hand, foot, digital, and toe also revealed significant reductions to HRQOL (Table 2). In the univariate analyses, greater and statistically significant reductions were seen in both univariate SF36PFS at 1 year (coefficient −4.7; 95% CI: −7.5, −1.9) and 2 years after incident fracture (coefficient −5.9; 95% CI: −9.2, −2.7). The latter also producing a clinically significant reduction. Significant reductions in SF36VS (coefficient −3.9; 95% CI: −6.2, −1.7) and a clinically significant reduction in EQ-5D (coefficient −0.05; 95% CI: −0.08, −0.02) were seen only at 1 year after incident fracture.

Previously mentioned multivariable adjustments were also conducted on analyses that excluded single region hand, foot, digital, and toe fractures. A statistically significant reduction in SF36PFS was seen at 2 years following an incident fracture (coefficient −3.8; 95% CI: −6.8, −0.8) (Table 2). Statistically significant reductions in SF36VS (coefficient −2.6; 95% CI: −5.2 to −0.1) and a clinically significant reduction in EQ-5D (coefficient −0.04; 95% CI: −0.07, −0.00) were seen only at 1 year following an incident fracture (Table 2). No significant differences in “excellent/very good/good” GH rating were found in those with and those without an incident fracture at all follow-up time periods.

Additional before and after analyses were conducted to examine changes in HRQOL measures in those incurring a single hip or vertebral fracture, those with a nonhip nonvertebral fracture, and also those with multiple regions fractures (Table 3). Changes to HRQOL irrespective of fracture location followed a similar trend to the previous multivariate analyses. However, these changes appeared to be more pronounced in those with a hip or vertebral fracture and also in those with a single incident fracture event occurring in multiple locations (Table 3). Significant and clinically important reductions in EQ-5D were also observed 1 year after incident fracture in women with a previous fracture history after the age of 45 years and who had also suffered an incident fracture in one location (data not shown).

## 4. Discussion

In this prospective study, data collected immediately prior to as well as in the years following an incident fracture were analysed to ascertain impacts to HRQOL succeeding the fracture. To our knowledge, this approach of using longitudinal data has not been previously conducted to a great extent among relatively young and healthy community-based postmenopausal women. In light of this, certain comparisons of the current results will be made to studies which relied on retrospective recollections of baseline HRQOL.

In past literature, osteoporosis-related fractures equated to fractures resulting from low impact trauma such as falling from a standing height [18]. Major trauma fractures which have been less commonly associated with fragility fractures in the literature included those incurred through motor vehicle accidents or falls greater than standing height (excluding falls from stairs) [18, 19]. Previous studies, however, have found that the association with osteoporosis and the risk for subsequent fractures are similar regardless of whether fractures occurred as a result of low or major impact trauma [19–23]. In consideration of this, the results of this analysis of HRQOL included fractures irrespective of the degree of impact trauma. However, subanalyses were conducted excluding minor fractures occurring at the hand, foot, fingers, and toes to determine HRQOL effects in common osteoporosis-associated fracture sites.

In the current study, clinically important differences were seen in EQ-5D 1 year after incident fracture (Table 2). These reductions were particularly evident in those incurring fractures in the hip or spine and also in those sustaining a single incident fracture event occurring in multiple locations (Table 3). These findings concur with literature presenting the detrimental effect on EQ-5D following an incident fracture. In a recent Australian study of 915 adults aged ≥50 years (mean age of 69.8 years), Abimanyi-Ochom et al. reported clinically important differences in EQ-5D scores at 12 months after incident fracture [24]. In the same study, hip and vertebral fractures were associated with the greatest clinically significant differences in EQ-5D scores in Australian adults 12 months after fracture [24]. In another study of 635 Swedish patients with a similar age range as the GLOW study (age range of 50–96 years), hip fractures decreased EQ-5D at 1 year after fracture compared to perceived prefracture scores [12]. Hagino and colleagues also described the greatest reduction in 1 year after hip fracture EQ-5D compared to other fracture sites (excluding vertebral fractures) in their prospective study of women aged ≥45 years [25]. As the cohort of postmenopausal women in the current study may be considered to be “relatively” young (age: median 65; interquartile range: 60–73 years), these findings highlight that reductions to HRQOL not only are limited to “elderly” populations but also affect somewhat “young” postmenopausal women with unchanged ratings of good general health following an incident fracture.

Statistically significant reductions in SF36VS were noted 1 year after incident fracture when considering all fracture types as well as excluding hand, foot, digital, and toe fractures (Table 2). These reductions, however, were not considered to be clinically important [16]. Similar changes to incident fractures on SF36VS 1 year after incident fracture have been previously described in “younger” cohorts with a comparable age range to the women in the current study. Hallberg et al. described changes to SF36VS to be either clinically or statistically significant and even increased 2 years after fracture in a cohort of Swedish women (aged 55–75 years) with a new fracture [26]. The SF36VS “*was intended to tap both positive health states (e.g., energy) as well as somatic expressions of physical illness and psychological distress (e.g., fatigue)…*” [27]. The effects of incident fractures on SF36VS may have varied effects on differing age groups of women, with younger cohorts being possibly less affected by reductions to SF36VS compared to older cohorts.

Statistically significant reductions to SF36PFS were also noted in the current sample at 2 years after incident fracture (Table 2), particularly in those sustaining hip or spine fractures (Table 3). Similar findings have been previously reported in the literature using retrospective recollections of prefracture scores [26, 28]. In a study of postmenopausal Swedish women (age range of 55–75 years), Hallberg et al. described reductions to SF36PFS 2 years after incident hip fracture [26]. Another prospective, longitudinal case-control study of patients aged ≥50 years (age range of 50–90 years), also found greater reductions in SF36PFS at 1 year after hip fracture. In this study, no further improvements were seen at 2 years after fracture, with SF36PFS scores plateauing at 1 year after hip fracture [28].

Reductions in HRQOL in younger populations may further contribute to the burden associated with osteoporotic fractures, particularly as refracture rates are higher in those with a prior fracture [29, 30]. In this study, significant and clinically important reductions in EQ-5D were seen in women with a previous history of fracture after the age of 45 years who had also suffered an incident fracture in one location. This is in agreement with findings reported from prior studies describing the detrimental effect prior fractures have on ED-5D following an incident fracture [12, 26]. In one study, prior fractures, particularly in the wrist, have been previously associated with significant reductions in EQ-5D in men and women aged >50 years [12]. Significantly reduced SF36PFS (*P* = 0.004) and SF36VS (*P* < 0.001) both at baseline and 2 years after incident fracture were also reported in women (age range of 55–75 years) with a previous fracture after the age of 40 years compared to those without a previous fracture [26]. The same study also reported hip fractures to have a greater impact on HRQOL compared to forearm and humeral fractures [26].

It is interesting to view the current results in light of the background characteristics of the sample population. Participants of the current study were recruited predominantly from higher socioeconomic locations in Sydney, Australia, where the majority (77%) had achieved at least a Higher School Certificate (12 years of study) contrasted to the 65% of those attaining a similar level of education in the greater Sydney area [31, 32]. The majority of women in the current sample (95%) also reported having private health insurance, contrasted by 52% of women aged ≥55 years in the Australian population [33, 34]. When considering this, it might be presumed that women of this study would be more likely to access treatment and rehabilitation following a fracture. After sustaining an incident fracture, 68% (138/204) sought medical advice from a doctor; 57% (120/212) sought medical treatment in a hospital with 29% (55/189) needing surgery and 19% (39/209) requiring further rehabilitation. In the current study, nearly 37.5% of those reporting an incident fracture also reported having a prior fracture after the age of 45 with only 18.1% being on AOM prior to an incident fracture occurring. In addition, despite originally seeking medical advice after fracture, the antiosteoporosis treatment rate after fracture (46.2%) in this current sample is low in those with both a previous fracture history and a consequent incident fracture. These findings highlight a possible unmet need for rehabilitation and treatment following an incident fracture in this population that may be present in similar groups. The detrimental impact of previous fractures on HRQOL coupled with below optimal treatment rates also accentuates the need for more proactive preventive measures particularly in “younger” postmenopausal women [26].

This study has limitations that should be considered before interpreting the findings. Participants were recruited predominantly from higher socioeconomic backgrounds, attaining higher education levels and having greater access to private health insurance compared to the Greater Sydney area. The results presented may not be generalizable to women in other countries. Future studies in more diverse populations of women, including “older” cohorts of elderly women, may be needed to further explore changes to HRQOL. Future studies should also explore the possible unmet need for rehabilitation and treatment following an incident fracture in this population that may be present in varying groups. Analyses in the study were also restricted by a limited number of fractures (*n* = 266). Of these fractures, only 28 were located at the hip or vertebral regions and numerous minor fractures were located in the hand, foot, fingers or toes (*n* = 66). Additionally, data on incident fractures were obtained through participant self-report with the authors unable to conduct radiographic verification. While possible misclassification or misreporting of fractures may have occurred and subclinical fractures may not have been reported, other studies have shown that self-reported fracture rates are relatively accurate [35–37].

This is a large longitudinal prospective study of Australian, community-based postmenopausal women examining the effects on HRQOL following an incident fracture. Although this is not the first study to explore HRQOL after fracture, it is one of a few large prospective studies collecting data immediately prior to as well as after an incident fracture to examine the true impact of incident fractures on HRQOL. The results in this current study extend and enrich data presented from previous cross-sectional studies as well as prospective studies using retrospective recollections for HRQOL.

In conclusion, fracture incidence reduced HRQOL among relatively young and healthy postmenopausal women and the reduction in European Quality of Life measure was clinically important. These reductions were seen particularly in those sustaining incident fractures in the hip and spine regions. The findings highlight the need for including HRQOL measures in evaluation of the risk-benefit profiles of osteoporosis medications as well as fracture prevention programs.

## Figures and Tables

**Table 1:** Baseline (first assessment) characteristics of the study women.

	Number	Incident fracture during the follow-up		*P* value
		No (*n* = 2,606)	Yes (*n* = 266)	
Age (years), mean (standard deviation (sd))	2,872	66.8 (8.8)	67.3 (9.2)	0.36
Body mass index (kg/m^2^), mean (sd)	2,615	25.9 (5.3)	26.0 (5.1)	0.77
Private health insurance, *n* (%)	2,843	2,480 (96.1)	251 (95.8)	0.82
Tertiary education, *n* (%)	2,827	1,202 (46.8)	126 (49.0)	0.49
Prior year weight loss (≥5 kgs), *n* (%)	2,839	169 (6.6)	17 (6.5)	0.99
Current smoking, *n* (%)	2,855	117 (4.5)	21 (7.9)	0.01
Alcohol drinking (≥7 drinks/week), *n* (%)	2,858	884 (34.1)	95 (36.1)	0.50
Prior fracture after 45 years, *n* (%)	2,834	604 (23.5)	97 (37.5)	<0.001
Prior fracture after 45 years and being on treatment, *n* (%)^#^	2,807	259 (10.0)	46 (18.1)	<0.001
Any comorbidity^∧^, *n* (%)	2,808	1,548 (60.7)	179 (69.9)	0.004
Number of falls in the prior year, *n* (%)	2,852			0.04
0		1,633 (63.1)	148 (56.1)	
1		631 (24.4)	71 (26.9)	
2		324 (12.5)	45 (17.1)	

^#^Prior fracture after the age of 45 years and being on treatment for osteoporosis at baseline (first assessment). Treatment was defined as self-reported use of antiosteoporosis medications (i.e., oestrogen, selective oestrogen receptor modulators, bisphosphonates, calcitonin, parathyroid hormone, and strontium).

^∧^Ever diagnosed with asthma, chronic bronchitis/emphysema, osteoarthritis/degenerative joint disease, rheumatoid arthritis, high cholesterol, hypertension, or osteoporosis.

**Table 2:** Impacts of incident fracture on health related quality of life measures and general health.

Assessment	SF-36 physical function score		SF-36 vitality scale		Quality of life (EQ-index)		General health (≥good)	
	Coefficient (95% CI)		Coefficient (95% CI)		Coefficient (95% CI)		Odds ratio (95% CI)	
	All fractures included	Minor fractures excluded^§^	All fractures included	Minor fractures excluded^§^	All fractures included	Minor fractures excluded^§^	All fractures included	Minor fractures excluded^§^
*Univariate analysis *								
Prior to incident fracture	0	0	0	0	0	0	1.00	1.00
The 1st year after incident fracture	−3.3^*∗∗*^ (−5.5 to −1.0)	−4.7^*∗∗∗*^ (−7.5 to −1.9)	−4.1^*∗∗∗*^ (−6.0 to −2.1)	−3.9^*∗∗∗*^ (−6.2 to −1.7)	−0.04^*∗∗∗*^ (−0.07 to −0.02)	−0.05^*∗∗∗*^ (−0.08 to −0.02)	0.80^*∗*^ (0.67 to 0.96)	0.78 (0.58 to 1.05)
The 2nd year after incident fracture	−5.0^*∗∗∗*^ (−7.6 to −2.3)	−5.9^*∗∗∗*^ (−9.2 to −2.7)	−0.9 (−3.0 to 1.2)	−0.4 (−2.7 to 2.0)	−0.01 (−0.03 to 0.01)	−0.01 (−0.04 to 0.02)	0.86 (0.69 to 1.07)	0.88 (0.60 to 1.28)
The 3rd year after incident fracture	−3.7 (−7.6 to 0.1)	−3.2 (−7.3 to 1.0)	−1.1 (−4.3 to 2.2)	−0.7 (−4.5 to 3.1)	−0.03 (−0.06 to 0.01)	−0.04 (−0.09 to 0.00)	0.97 (0.71 to 1.32)	0.72 (0.41 to 1.26)

*Multivariable analysis * ^∧^								
Prior to incident fracture	0	0	0	0	0	0	1.00	1.00
The 1st year after incident fracture	−1.6 (−4.2 to 1.0)	−2.9 (−6.1 to 0.3)	−3.0^*∗∗*^ (−5.1 to −0.8)	−2.6^*∗∗*^ (−5.2 to −0.1)	−0.03^*∗*^ (−0.06 to −0.00)	−0.04^*∗*^ (−0.07 to −0.00)	0.97 (0.78 to 1.21)	0.95 (0.66 to 1.38)
The 2nd year after incident fracture	−3.0^*∗*^ (−5.6 to −0.5)	−3.8^*∗∗*^ (−6.8 to −0.8)	−0.8 (−3.2 to 1.6)	0.03 (−2.6 to 2.7)	−0.02 (−0.04 to 0.01)	−0.02 (−0.05 to 0.01)	0.92 (0.70 to 1.19)	1.10 (0.69 to 1.73)
The 3rd year after incident fracture	0.2 (−4.1 to 4.4)	1.5 (−2.6 to 5.6)	0.6 (−3.1 to 4.3)	1.2 (−3.1 to 5.5)	−0.02 (−0.06 to 0.02)	−0.03 (−0.09 to 0.02)	1.04 (0.72 to 1.50)	0.84 (0.44 to 1.61)

^§^Minor fractures: hand, foot, toe, and digital fractures.

^∧^Adjusted for age, private insurance, tertiary education, history of prior fracture after 45 years, body mass index, prior year weight loss ≥5 kgs, any comorbidity, current smoking, drinking ≥7 drinks/week, and number of falls in the prior year.

^*∗*^ *P* value ≤0.05.

^*∗∗*^ *P* value ≤0.01.

^*∗∗∗*^ *P* value ≤0.001.

**Table 3:** Changes in health related quality of life measures according to fracture location.

Fracture type	Quality of life measure	Assessment time	No.	Mean	Mean difference from baseline (95% CI)
Hip or vertebral (*n* = 26)	SF-36 physical function score	Prior to incident fracture	26	49.8	0
		1 year after fracture	24	53.2	0.1 (−10.2, 10.4)
		2 years after fracture	13	56.2	−9.4 (−17.9, −0.9)^*∗*^
	SF-36 vitality scale	Prior to incident fracture	25	51.1	0
		1 year after fracture	24	48.9	−1.2 (−9.3, 6.9)
		2 years after fracture	12	62.5	1.2 (−7.8, 10.3)
	EQ-5D	Prior to incident fracture	25	0.67	0
		1 year after fracture	23	0.58	−0.10 (−0.19, −0.01)^*∗*^
		2 years after fracture	13	0.76	−0.03 (−0.12, 0.06)
	General health^§^	Prior to incident fracture	26	3.1	0
		1 year after fracture	25	3.4	0.36 (0.10, 0.62)^*∗∗*^
		2 years after fracture	13	2.9	0.23 (−0.13, 0.59)

Nonhip, nonvertebral (*n* = 212)	SF-36 physical function score	Prior to incident fracture	207	75.6	0
		1 year after fracture	200	72.9	−2.4 (−4.7, −0.0)^*∗*^
		2 years after fracture	132	72.9	−3.9 (−7.2, −0.7)
	SF-36 vitality scale	Prior to incident fracture	206	59.6	0
		1 year after fracture	199	56.5	−3.0 (−5.3, −0.7)^*∗*^
		2 years after fracture	129	61.3	−0.2 (−3.3, 3.0)
	EQ-5D	Prior to incident fracture	201	0.78	0
		1 year after fracture	197	0.77	−0.02 (−0.04, 0.01)
		2 years after fracture	125	0.80	0.01 (−0.02, 0.04)
	General health^§^	Prior to incident fracture	208	2.6	0
		1 year after fracture	206	2.6	0.06 (−0.03, 0.16)
		2 years after fracture	132	2.5	0.53 (−0.06, 0.17)

Multiple regions^∧^ (*n* = 28)	SF-36 physical function score	Prior to incident fracture	28	71.2	0
		1 year after fracture	27	63.0	−7.4 (−18.4, 3.7)
		2 years after fracture	18	63.1	−6.03 (−14.46, 2.39)
	SF-36 vitality scale	Prior to incident fracture	28	53.4	0
		1 year after fracture	28	49.6	−3.7 (−11.1, 3.6)
		2 years after fracture	18	53.8	−1.0 (−4.8, 2.7)
	EQ-5D	Prior to incident fracture	28	0.80	0
		1 year after fracture	27	0.67	−0.13 (−0.23, −0.03)^*∗*^
		2 years after fracture	17	0.75	−0.07 (−0.17, 0.03)
	General health^§^	Prior to incident fracture	28	2.7	0
		1 year after fracture	27	2.7	0.00 (−2.45, 2.45)
		2 years after fracture	19	2.7	0.00 (−0.32, 0.32)

^∧^An incident resulting fracture in multiple regions.

^*∗*^ *P* value ≤0.05.

^*∗∗*^ *P* value ≤0.01.

^§^Self-rated general health using 5-point Likert scales (excellent, very good, good, fair, or poor).
